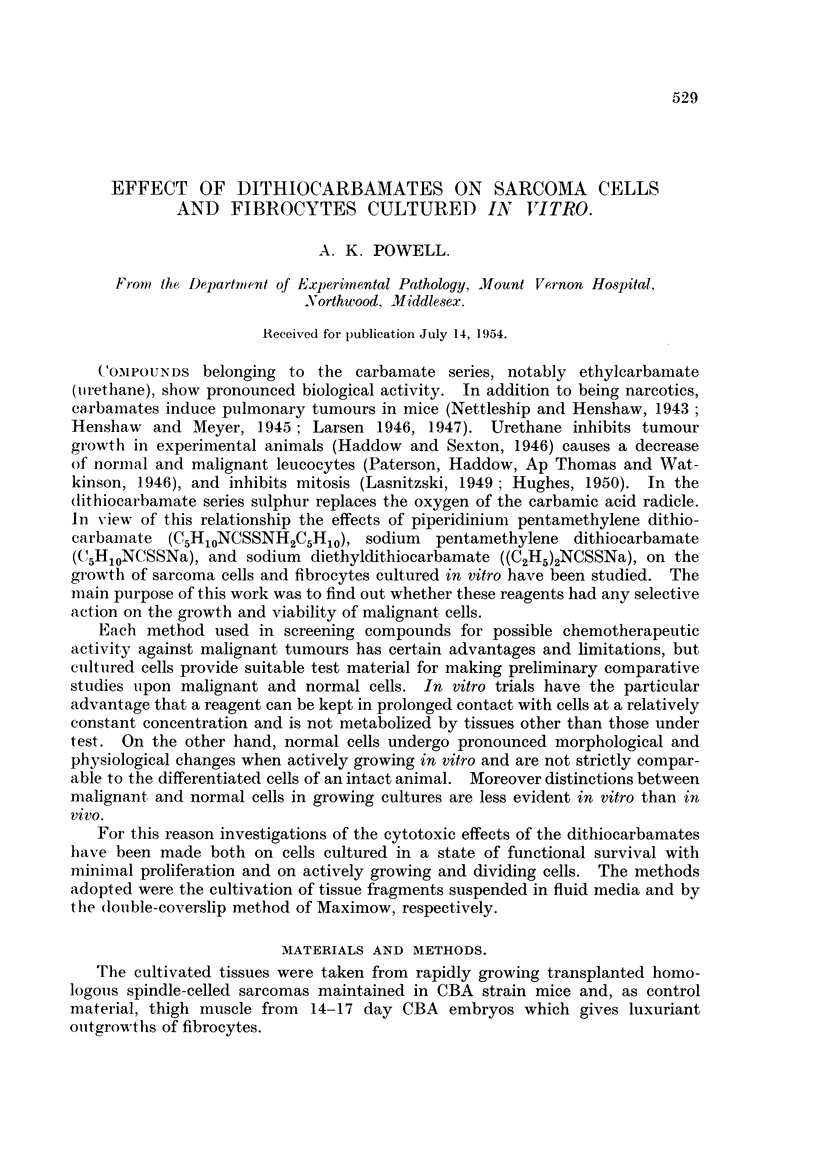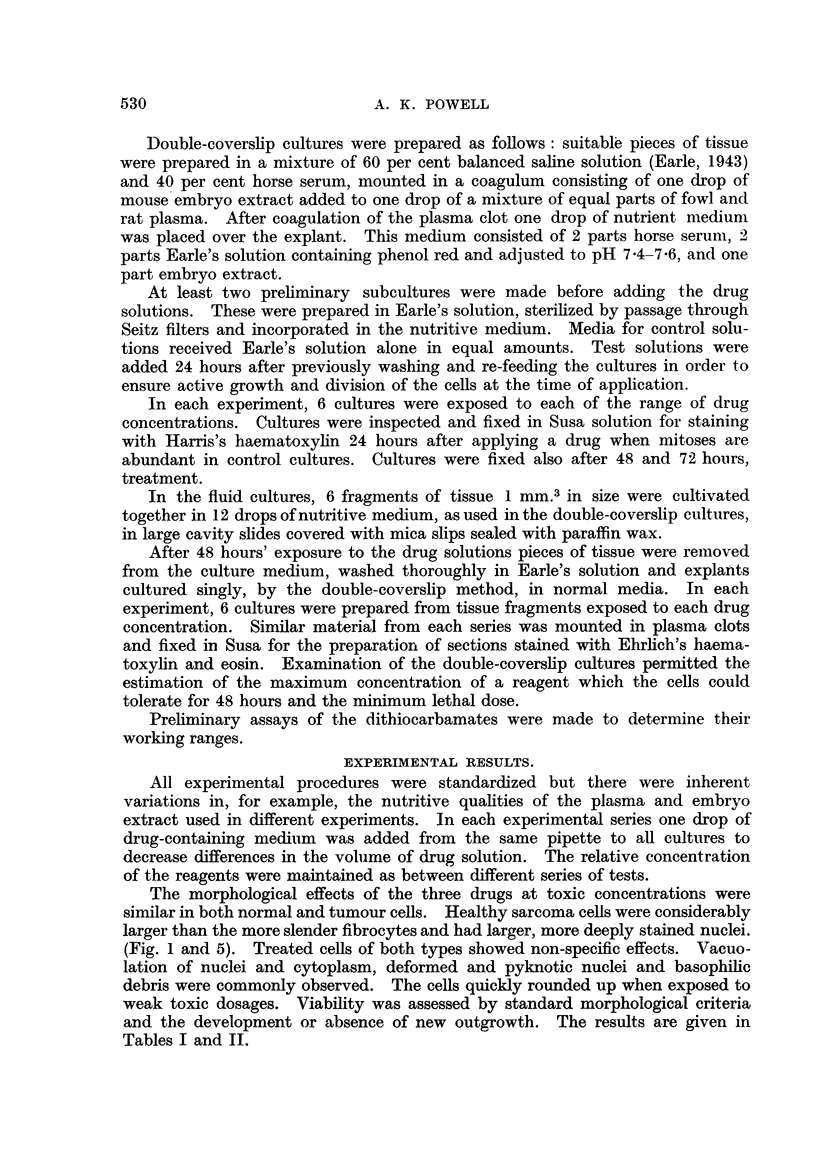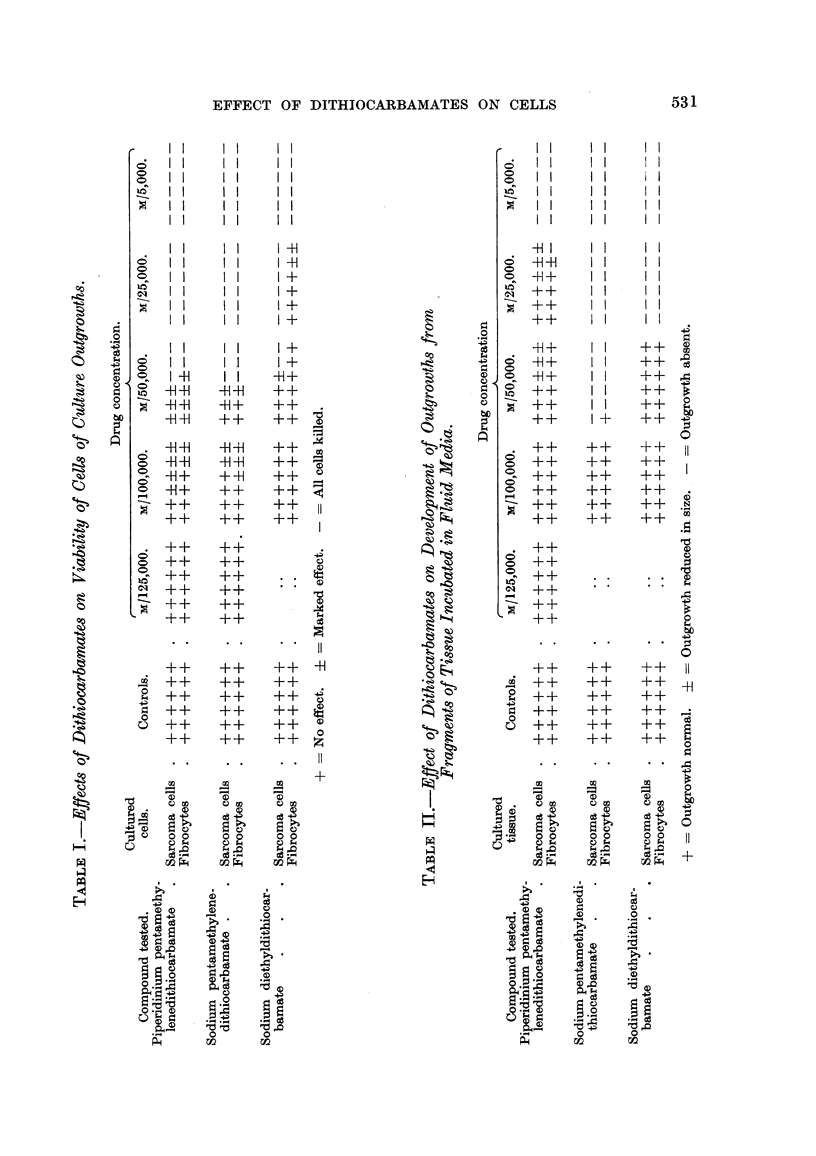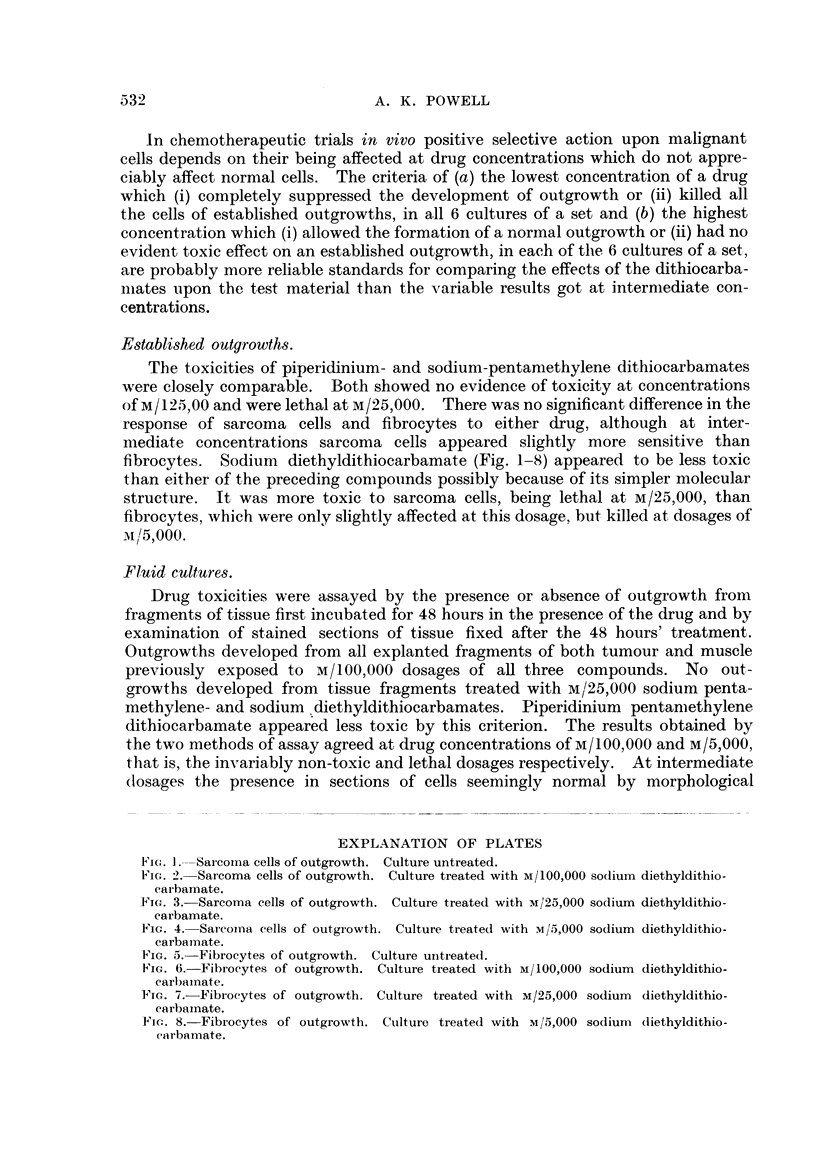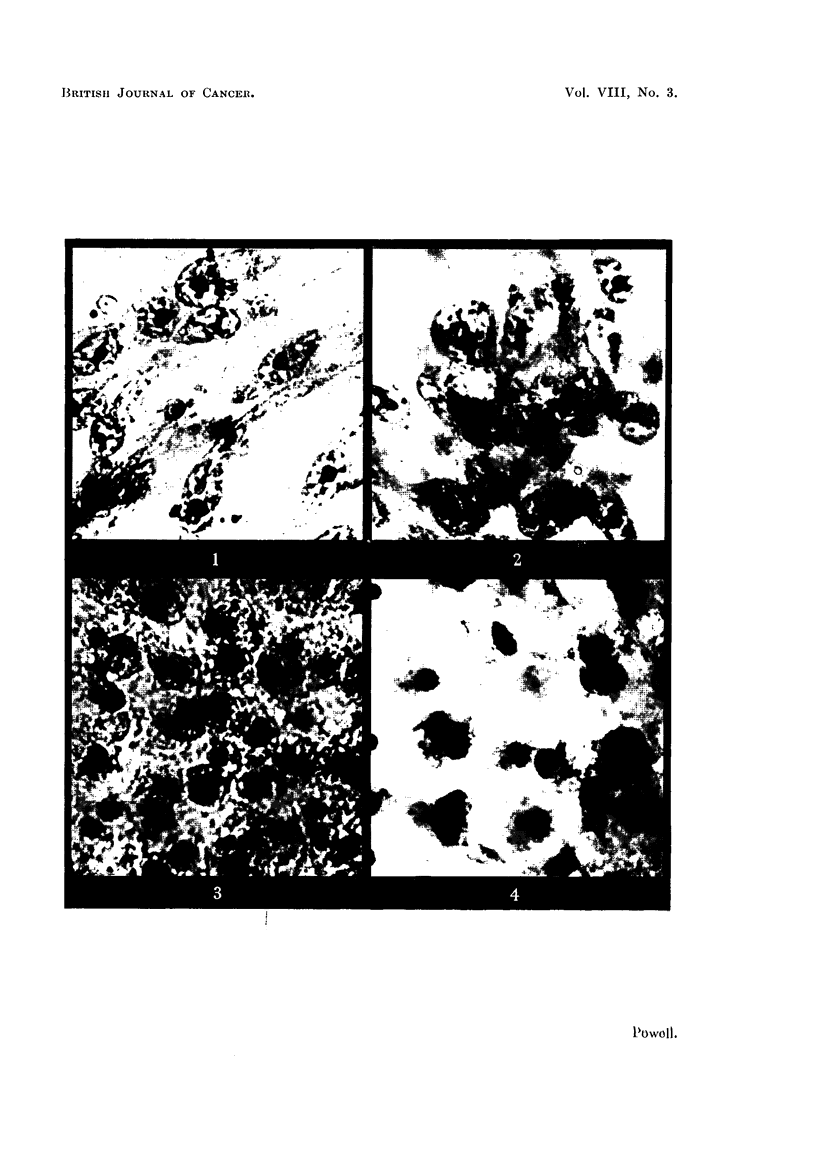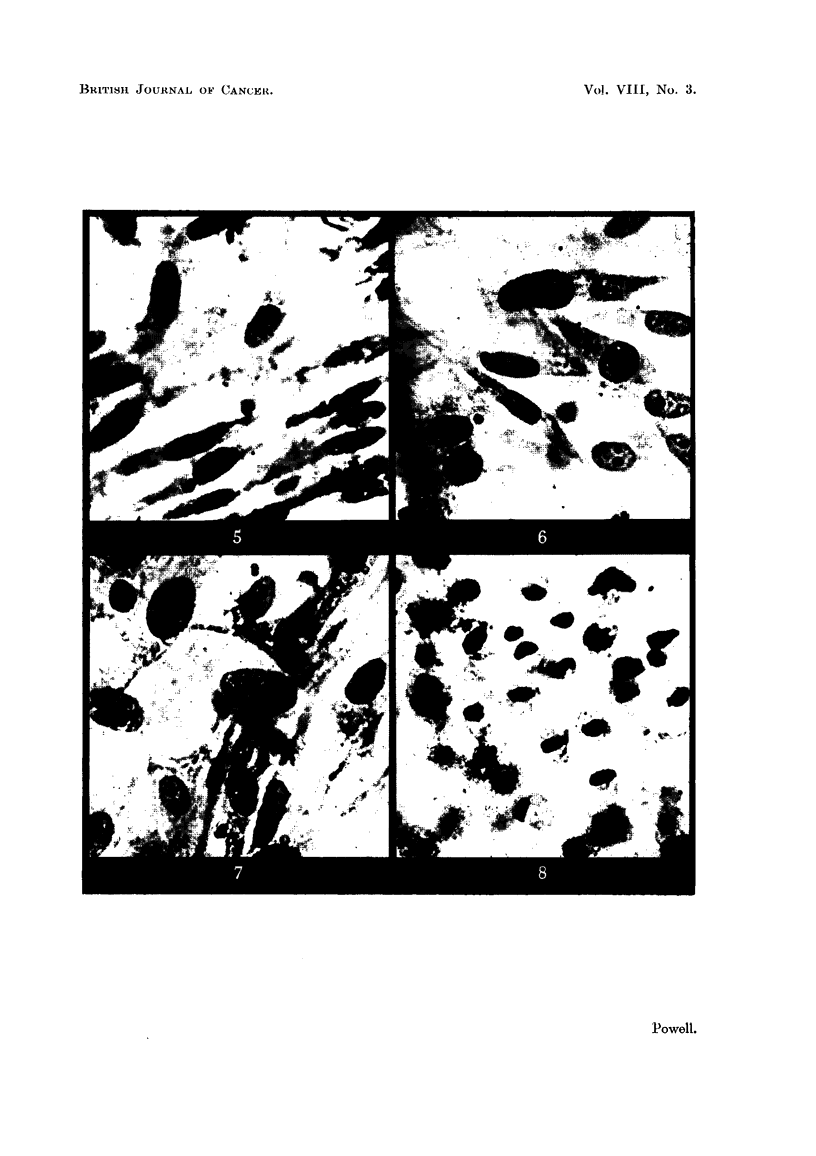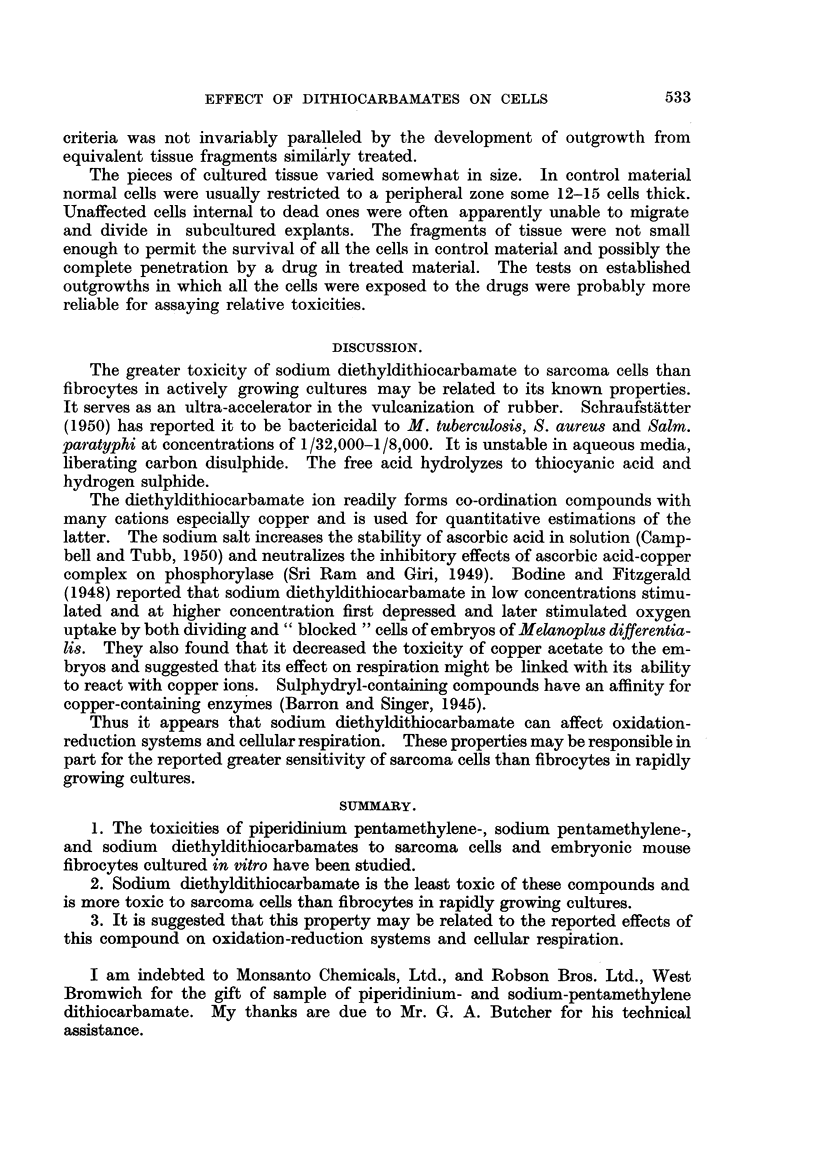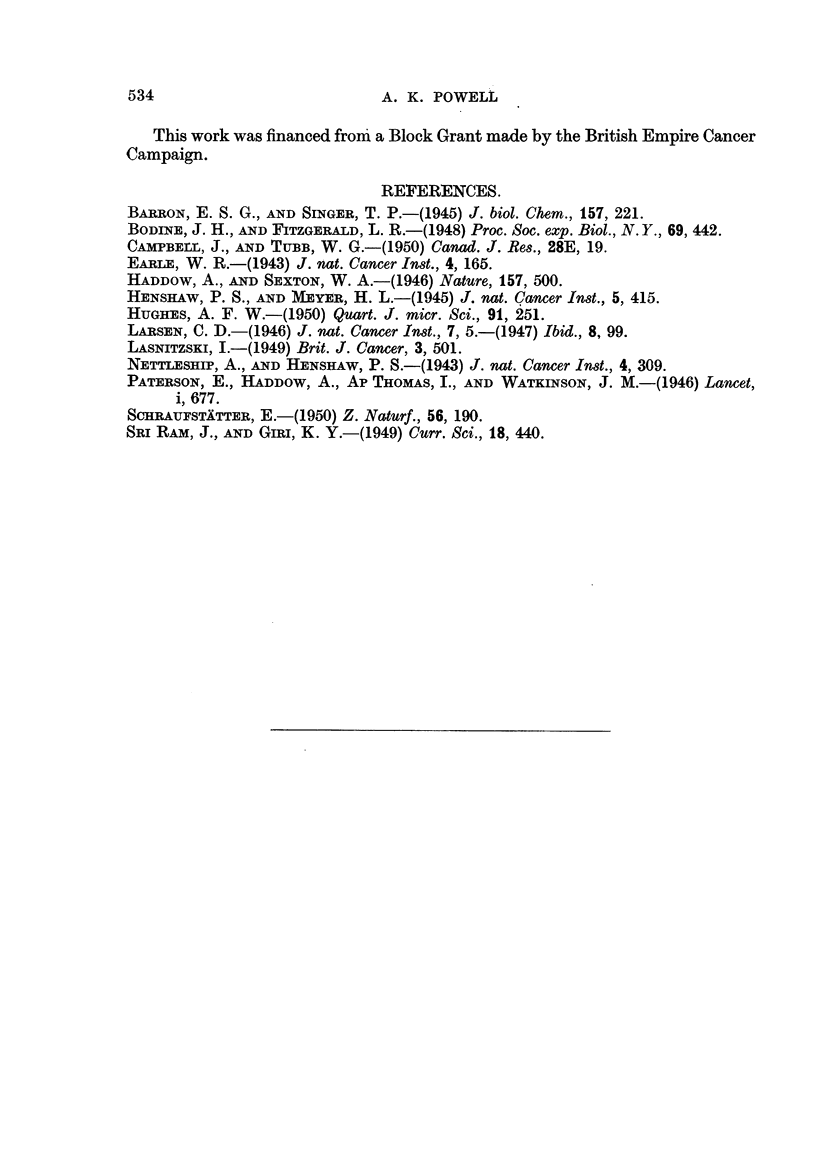# Effect of Dithiocarbamates on Sarcoma Cells and Fibrocytes Cultured In Vitro

**DOI:** 10.1038/bjc.1954.56

**Published:** 1954-09

**Authors:** A. K. Powell

## Abstract

**Images:**


### EFFECT OF DITHIOCARBAMATES ON SARCOMA CELLS AND FIBROCYTES CULTURED *IN VITRO*.

A. K. POWELL.

*From the Department of Experimental Pathology, Mount Vernon Hospital, Northwood, Middlesex.*

Received for publication July 14, 1954.

COMPOUNDS belonging to the carbamate series, notably ethylcarbamate (urethane), show pronounced biological activity. In addition to being narcotics, carbamates induce pulmonary tumours in mice (Nettleship and Henshaw, 1943 ; Henshaw and Meyer, 1945 ; Larsen 1946, 1947). Urethane inhibits tumour growth in experimental animals (Haddow and Sexton, 1946) causes a decrease of normal and malignant leucocytes (Paterson, Haddow, Ap Thomas and Watkinson, 1946), and inhibits mitosis (Lasnitzski, 1949 ; Hughes, 1950). In the dithiocarbamate series sulphur replaces the oxygen of the carbamic acid radicle. In view of this relationship the effects of piperidinium pentamethylene dithiocarbamate ($C_5H_{10}NCSSNH_2C_5H_{10}$), sodium pentamethylene dithiocarbamate ($C_5H_{10}NCSSNa$), and sodium diethyldithiocarbamate ($(C_2H_5)_2NCSSNa$), on the growth of sarcoma cells and fibrocytes cultured *in vitro* have been studied. The main purpose of this work was to find out whether these reagents had any selective action on the growth and viability of malignant cells.

Each method used in screening compounds for possible chemotherapeutic activity against malignant tumours has certain advantages and limitations, but cultured cells provide suitable test material for making preliminary comparative studies upon malignant and normal cells. *In vitro* trials have the particular advantage that a reagent can be kept in prolonged contact with cells at a relatively constant concentration and is not metabolized by tissues other than those under test. On the other hand, normal cells undergo pronounced morphological and physiological changes when actively growing *in vitro* and are not strictly comparable to the differentiated cells of an intact animal. Moreover distinctions between malignant and normal cells in growing cultures are less evident *in vitro* than *in vivo*.

For this reason investigations of the cytotoxic effects of the dithiocarbamates have been made both on cells cultured in a state of functional survival with minimal proliferation and on actively growing and dividing cells. The methods adopted were the cultivation of tissue fragments suspended in fluid media and by the double-coverslip method of Maximow, respectively.

### MATERIALS AND METHODS.

The cultivated tissues were taken from rapidly growing transplanted homologous spindle-celled sarcomas maintained in CBA strain mice and, as control material, thigh muscle from 14–17 day CBA embryos which gives luxuriant outgrowths of fibrocytes.

Double-coverslip cultures were prepared as follows : suitable pieces of tissue were prepared in a mixture of 60 per cent balanced saline solution (Earle, 1943) and 40 per cent horse serum, mounted in a coagulum consisting of one drop of mouse embryo extract added to one drop of a mixture of equal parts of fowl and rat plasma. After coagulation of the plasma clot one drop of nutrient medium was placed over the explant. This medium consisted of 2 parts horse serum, 2 parts Earle's solution containing phenol red and adjusted to pH 7·4–7·6, and one part embryo extract.

At least two preliminary subcultures were made before adding the drug solutions. These were prepared in Earle's solution, sterilized by passage through Seitz filters and incorporated in the nutritive medium. Media for control solutions received Earle's solution alone in equal amounts. Test solutions were added 24 hours after previously washing and re-feeding the cultures in order to ensure active growth and division of the cells at the time of application.

In each experiment, 6 cultures were exposed to each of the range of drug concentrations. Cultures were inspected and fixed in Susa solution for staining with Harris's haematoxylin 24 hours after applying a drug when mitoses are abundant in control cultures. Cultures were fixed also after 48 and 72 hours, treatment.

In the fluid cultures, 6 fragments of tissue 1 mm.$^3$ in size were cultivated together in 12 drops of nutritive medium, as used in the double-coverslip cultures, in large cavity slides covered with mica slips sealed with paraffin wax.

After 48 hours' exposure to the drug solutions pieces of tissue were removed from the culture medium, washed thoroughly in Earle's solution and explants cultured singly, by the double-coverslip method, in normal media. In each experiment, 6 cultures were prepared from tissue fragments exposed to each drug concentration. Similar material from each series was mounted in plasma clots and fixed in Susa for the preparation of sections stained with Ehrlich's haematoxylin and eosin. Examination of the double-coverslip cultures permitted the estimation of the maximum concentration of a reagent which the cells could tolerate for 48 hours and the minimum lethal dose.

Preliminary assays of the dithiocarbamates were made to determine their working ranges.

### EXPERIMENTAL RESULTS.

All experimental procedures were standardized but there were inherent variations in, for example, the nutritive qualities of the plasma and embryo extract used in different experiments. In each experimental series one drop of drug-containing medium was added from the same pipette to all cultures to decrease differences in the volume of drug solution. The relative concentration of the reagents were maintained as between different series of tests.

The morphological effects of the three drugs at toxic concentrations were similar in both normal and tumour cells. Healthy sarcoma cells were considerably larger than the more slender fibrocytes and had larger, more deeply stained nuclei. (Fig. 1 and 5). Treated cells of both types showed non-specific effects. Vacuolation of nuclei and cytoplasm, deformed and pyknotic nuclei and basophilic debris were commonly observed. The cells quickly rounded up when exposed to weak toxic dosages. Viability was assessed by standard morphological criteria and the development or absence of new outgrowth. The results are given in Tables I and II.

TABLE I.—*Effects of Dithiocarbamates on Viability of Cells of Culture Outgrowths.*

| Compound tested. | Cultured cells. | Controls. | Drug concentration. M/125,000. | M/100,000. | M/50,000. | M/25,000. | M/5,000. |
|---|---|---|---|---|---|---|---|
| Piperidinium pentamethylenedithiocarbamate | Sarcoma cells | ++++++ | ++++++ | ++±±±± | ±±±––– | –––––– | –––––– |
| | Fibrocytes | ++++++ | ++++++ | ++++±± | ±±±±–– | –––––– | –––––– |
| Sodium pentamethylenedithiocarbamate | Sarcoma cells | ++++++ | ++++++ | ++++±± | +±±––– | –––––– | –––––– |
| | Fibrocytes | ++++++ | ++++++. | +++±±± | ++±––– | –––––– | –––––– |
| Sodium diethyldithiocarbamate | Sarcoma cells | ++++++ | .. | ++++++ | +++±–– | –––––– | –––––– |
| | Fibrocytes | ++++++ | .. | ++++++ | ++++++ | ++++±± | –––––– |

\+ = No effect. ± = Marked effect. – = All cells killed.

TABLE II.—*Effect of Dithiocarbamates on Development of Outgrowths from Fragments of Tissue Incubated in Fluid Media.*

| Compound tested. | Cultured tissue. | Controls. | Drug concentration M/125,000. | M/100,000. | M/50,000. | M/25,000. | M/5,000. |
|---|---|---|---|---|---|---|---|
| Piperidinium pentamethylenedithiocarbamate | Sarcoma cells | ++++++ | ++++++ | ++++++ | +++±±± | +++±±± | –––––– |
| | Fibrocytes | ++++++ | ++++++ | ++++++ | ++++++ | ++++±– | –––––– |
| Sodium pentamethylenedithiocarbamate | Sarcoma cells | ++++++ | .. | ++++++ | –––––– | –––––– | –––––– |
| | Fibrocytes | ++++++ | .. | ++++++ | +––––– | –––––– | –––––– |
| Sodium diethyldithiocarbamate | Sarcoma cells | ++++++ | .. | ++++++ | ++++++ | –––––– | –––––– |
| | Fibrocytes | ++++++ | .. | ++++++ | ++++++ | –––––– | –––––– |

\+ = Outgrowth normal. ± = Outgrowth reduced in size. – = Outgrowth absent.

In chemotherapeutic trials *in vivo* positive selective action upon malignant cells depends on their being affected at drug concentrations which do not appreciably affect normal cells. The criteria of (*a*) the lowest concentration of a drug which (i) completely suppressed the development of outgrowth or (ii) killed all the cells of established outgrowths, in all 6 cultures of a set and (*b*) the highest concentration which (i) allowed the formation of a normal outgrowth or (ii) had no evident toxic effect on an established outgrowth, in each of the 6 cultures of a set, are probably more reliable standards for comparing the effects of the dithiocarbamates upon the test material than the variable results got at intermediate concentrations.

*Established outgrowths.*

The toxicities of piperidinium- and sodium-pentamethylene dithiocarbamates were closely comparable. Both showed no evidence of toxicity at concentrations of M/125,00 and were lethal at M/25,000. There was no significant difference in the response of sarcoma cells and fibrocytes to either drug, although at intermediate concentrations sarcoma cells appeared slightly more sensitive than fibrocytes. Sodium diethyldithiocarbamate (Fig. 1–8) appeared to be less toxic than either of the preceding compounds possibly because of its simpler molecular structure. It was more toxic to sarcoma cells, being lethal at M/25,000, than fibrocytes, which were only slightly affected at this dosage, but killed at dosages of M/5,000.

*Fluid cultures.*

Drug toxicities were assayed by the presence or absence of outgrowth from fragments of tissue first incubated for 48 hours in the presence of the drug and by examination of stained sections of tissue fixed after the 48 hours' treatment. Outgrowths developed from all explanted fragments of both tumour and muscle previously exposed to M/100,000 dosages of all three compounds. No outgrowths developed from tissue fragments treated with M/25,000 sodium pentamethylene- and sodium diethyldithiocarbamates. Piperidinium pentamethylene dithiocarbamate appeared less toxic by this criterion. The results obtained by the two methods of assay agreed at drug concentrations of M/100,000 and M/5,000, that is, the invariably non-toxic and lethal dosages respectively. At intermediate dosages the presence in sections of cells seemingly normal by morphological

EXPLANATION OF PLATES

Fig. 1.—Sarcoma cells of outgrowth. Culture untreated.
Fig. 2.—Sarcoma cells of outgrowth. Culture treated with M/100,000 sodium diethyldithiocarbamate.
Fig. 3.—Sarcoma cells of outgrowth. Culture treated with M/25,000 sodium diethyldithiocarbamate.
Fig. 4.—Sarcoma cells of outgrowth. Culture treated with M/5,000 sodium diethyldithiocarbamate.
Fig. 5.—Fibrocytes of outgrowth. Culture untreated.
Fig. 6.—Fibrocytes of outgrowth. Culture treated with M/100,000 sodium diethyldithiocarbamate.
Fig. 7.—Fibrocytes of outgrowth. Culture treated with M/25,000 sodium diethyldithiocarbamate.
Fig. 8.—Fibrocytes of outgrowth. Culture treated with M/5,000 sodium diethyldithiocarbamate.

Powell.

Powell.

criteria was not invariably paralleled by the development of outgrowth from equivalent tissue fragments similarly treated.

The pieces of cultured tissue varied somewhat in size. In control material normal cells were usually restricted to a peripheral zone some 12–15 cells thick. Unaffected cells internal to dead ones were often apparently unable to migrate and divide in subcultured explants. The fragments of tissue were not small enough to permit the survival of all the cells in control material and possibly the complete penetration by a drug in treated material. The tests on established outgrowths in which all the cells were exposed to the drugs were probably more reliable for assaying relative toxicities.

### DISCUSSION.

The greater toxicity of sodium diethyldithiocarbamate to sarcoma cells than fibrocytes in actively growing cultures may be related to its known properties. It serves as an ultra-accelerator in the vulcanization of rubber. Schraufstätter (1950) has reported it to be bactericidal to *M. tuberculosis*, *S. aureus* and *Salm. paratyphi* at concentrations of 1/32,000–1/8,000. It is unstable in aqueous media, liberating carbon disulphide. The free acid hydrolyzes to thiocyanic acid and hydrogen sulphide.

The diethyldithiocarbamate ion readily forms co-ordination compounds with many cations especially copper and is used for quantitative estimations of the latter. The sodium salt increases the stability of ascorbic acid in solution (Campbell and Tubb, 1950) and neutralizes the inhibitory effects of ascorbic acid-copper complex on phosphorylase (Sri Ram and Giri, 1949). Bodine and Fitzgerald (1948) reported that sodium diethyldithiocarbamate in low concentrations stimulated and at higher concentration first depressed and later stimulated oxygen uptake by both dividing and " blocked " cells of embryos of *Melanoplus differentialis*. They also found that it decreased the toxicity of copper acetate to the embryos and suggested that its effect on respiration might be linked with its ability to react with copper ions. Sulphydryl-containing compounds have an affinity for copper-containing enzymes (Barron and Singer, 1945).

Thus it appears that sodium diethyldithiocarbamate can affect oxidation-reduction systems and cellular respiration. These properties may be responsible in part for the reported greater sensitivity of sarcoma cells than fibrocytes in rapidly growing cultures.

### SUMMARY.

1. The toxicities of piperidinium pentamethylene-, sodium pentamethylene-, and sodium diethyldithiocarbamates to sarcoma cells and embryonic mouse fibrocytes cultured *in vitro* have been studied.

2. Sodium diethyldithiocarbamate is the least toxic of these compounds and is more toxic to sarcoma cells than fibrocytes in rapidly growing cultures.

3. It is suggested that this property may be related to the reported effects of this compound on oxidation-reduction systems and cellular respiration.

I am indebted to Monsanto Chemicals, Ltd., and Robson Bros. Ltd., West Bromwich for the gift of sample of piperidinium- and sodium-pentamethylene dithiocarbamate. My thanks are due to Mr. G. A. Butcher for his technical assistance.

This work was financed from a Block Grant made by the British Empire Cancer Campaign.

### REFERENCES.

BARRON, E. S. G., AND SINGER, T. P.—(1945) *J. biol. Chem.*, **157**, 221.
BODINE, J. H., AND FITZGERALD, L. R.—(1948) *Proc. Soc. exp. Biol., N.Y.*, **69**, 442.
CAMPBELL, J., AND TUBB, W. G.—(1950) *Canad. J. Res.*, **28E**, 19.
EARLE, W. R.—(1943) *J. nat. Cancer Inst.*, **4**, 165.
HADDOW, A., AND SEXTON, W. A.—(1946) *Nature*, **157**, 500.
HENSHAW, P. S., AND MEYER, H. L.—(1945) *J. nat. Cancer Inst.*, **5**, 415.
HUGHES, A. F. W.—(1950) *Quart. J. micr. Sci.*, **91**, 251.
LARSEN, C. D.—(1946) *J. nat. Cancer Inst.*, **7**, 5.—(1947) *Ibid.*, **8**, 99.
LASNITZSKI, I.—(1949) *Brit. J. Cancer*, **3**, 501.
NETTLESHIP, A., AND HENSHAW, P. S.—(1943) *J. nat. Cancer Inst.*, **4**, 309.
PATERSON, E., HADDOW, A., AP THOMAS, I., AND WATKINSON, J. M.—(1946) *Lancet*, i, 677.
SCHRAUFSTÄTTER, E.—(1950) *Z. Naturf.*, **56**, 190.
SRI RAM, J., AND GIRI, K. Y.—(1949) *Curr. Sci.*, **18**, 440.